# Apple Juice Fermented with *Lactiplantibacillus plantarum* Improves Its Flavor Profile and Probiotic Potential

**DOI:** 10.3390/foods14132373

**Published:** 2025-07-04

**Authors:** Boqian Zhou, Zhuobin Xing, Yiting Wang, Xin Guan, Fuyi Wang, Jiaqi Yin, Zhibo Li, Qiancheng Zhao, Hongman Hou, Xue Sang

**Affiliations:** 1 College of Food Science and Engineering, Dalian Ocean University, Dalian 116023, China; zhouboqian00@163.com (B.Z.); wangyiting714@163.com (Y.W.); 18741821172@163.com (J.Y.); lzb@dlou.edu.cn (Z.L.); qczhao@dlou.edu.cn (Q.Z.); 2 School of Food Science and Technology, Dalian Polytechnic University, Dalian 116034, China; houhongman@dlpu.edu.cn; 3 Dalian Key Laboratory of Marine Bioactive Substances Development and HighValue Utilization, Dalian 116023, China; 4 Liaoning Provincial Marine Healthy Food Engineering Research Centre, Dalian 116000, China; 5 Collaborative Innovation Center of Provincial and Ministerial Co-Construction for Marine Food Deep Processing, Dalian Polytechnic University, Dalian 116034, China

**Keywords:** obesity, *Lactiplantibacillus plantarum*, fermented apple juice, flavor, gut microbiota

## Abstract

Fermented apple juice (FAJ), a nutrient-dense beverage rich in vitamins, offers multiple health benefits, including improved digestion, enhanced fat metabolism, and sustained energy provision with reduced caloric intake. To advance the development of probiotic-enriched flavored and functional juices, this study establishes *Lactiplantibacillus plantarum* (*L. plantarum*) as a safe and effective starter culture for apple juice fermentation. The selected strain exhibited minimal biogenic amine synthesis, producing only 30.55 ± 1.2 mg/L of putrescine and 0.59 ± 0.55 mg/L of cadaverine, while histamine and tyramine were undetectable. Furthermore, the strain demonstrated no hemolytic activity and exhibited robust biofilm-forming capacity, reinforcing its suitability for fermentation applications. An electronic nose analysis revealed that *L. plantarum* significantly enriched the volatile compound profile of FAJ, leading to an improved flavor profile. The strain also displayed excellent growth adaptability in the apple juice matrix, further optimizing fermentation efficiency and sensory quality. Crucially, 16S rRNA sequencing demonstrated that FAJ specifically restructures the gut microbiota in obese individuals, significantly elevating the relative abundance of beneficial genera, including *Enterococcus*, *Parabacteroides*, and *Bifidobacterium* (*p* < 0.05). Concurrently, FAJ enhanced glycolytic activity, suggesting a potential role in metabolic regulation. Collectively, these findings confirm that *L. plantarum*-fermented FAJ combines favorable sensory properties and safety with promising anti-obesity effects mediated through gut microbiome modulation and metabolic pathway activation. This study provides a critical scientific foundation for designing next-generation functional fermented beverages with targeted health benefits.

## 1. Introduction

Obesity is recognized as a persistent metabolic disorder resulting from the interplay of genetic, biological, environmental, behavioral, socio-cultural, and economic factors [1]. Recently, China’s National Health Commission launched a three-year initiative, “Weight Management Year,” targeting the general public, highlighting that obesity—defined by excessive adipose tissue accumulation (BMI ≥ 28 kg/m^2^)—has become a major global public health concern [2]. While non-modifiable factors such as genetics contribute to obesity pathogenesis, modifiable dietary factors present promising intervention targets [3,4]. Emerging evidence suggests that increased consumption of phytochemical-rich foods could help mitigate obesity and related metabolic disorders via diverse biological pathways, such as regulating cellular energy homeostasis, enhancing microbial community balance in the gastrointestinal tract, attenuating inflammatory responses, and stimulating thermogenic activation in white adipose tissue [5], driving interest in developing novel functional food products.

The expert consensus panel defines probiotics as “live microorganisms that, when administered in adequate amounts, confer a defined health benefit on the host” [6]. These beneficial microorganisms exert their effects through diverse metabolic activities, breaking down macromolecules, including proteins, carbohydrates, and fats, in food substrates. Among various probiotic strains, *L. plantarum* has demonstrated particular promise due to its GRAS status and versatile fermentation capabilities [7,8]. Through sophisticated quorum-sensing mechanisms and genetic regulation, this microorganism actively participates in essential metabolic processes, including amino acid biosynthesis and glycolytic pathways, yielding diverse flavor-active compounds, such as esters, aldehydes, and ketones [9,10]. *L. plantarum* has been successfully employed in various food matrices, ranging from traditional dairy products to innovative plant-based fermentations, such as wolfberry juice and chickpea milk [11,12]. The fermentation process not only enhances food preservation but also improves nutritional quality through microbial bioconversion of macromolecules. With increasing consumer awareness of dairy-related health concerns, these non-dairy fermented alternatives have emerged as attractive functional food options [13], with their characteristic flavor profiles resulting from precisely these microbial metabolic activities.

Apples contain abundant bioactive compounds, including polyphenols, vitamins, and dietary fiber, that provide both nutritional value and fermentation substrates [14]. These components make apple juice an excellent probiotic fermentation medium, offering readily available carbohydrates for microbial growth. However, conventional processing methods like pasteurization may degrade heat-sensitive nutrients [15]. Controlled fermentation with selected LAB strains presents an effective alternative, enhancing both functionality and sensory properties while preserving nutritional quality [13]. With growing consumer awareness of dairy-related concerns, such as lactose intolerance, dairy allergies, and cholesterol-associated health risks, there is increasing interest in non-dairy fermented alternatives, including fruit-based probiotic beverages. Specifically, strains from traditional fermentation ecosystems are preferred as they are adapted to fruit matrices and can ensure safety while developing desirable flavor profiles. The fermentation process transforms apple juice into a functional beverage with enhanced bioactivity and probiotic benefits, addressing key challenges in developing non-dairy fermented products. This approach maintains the inherent nutritional advantages of apples while overcoming limitations of conventional juice processing methods.

Flavor development represents a crucial quality parameter in fermented beverages, typically assessed through sensory analysis [16]. Among probiotic strains used for apple juice fermentation, *L. plantarum* demonstrates superior performance, exhibiting not only enhanced viability and elevated superoxide dismutase (SOD) activity but also significant flavor-modulating capabilities [17,18]. During fermentation, *L. plantarum* generates diverse volatile compounds (including alcohols, ketones, aldehydes, and acids) while simultaneously reducing undesirable flavor components, thereby improving the overall sensory profile of fermented apple juice (FAJ) [19,20]. Beyond sensory attributes, *L. plantarum*-FAJ exhibits notable metabolic benefits, including the regulation of lipid metabolism through the modulation of cholesterol profiles and maintenance of gut microbiota homeostasis, suggesting potential applications in obesity management [21,22]. These effects align with emerging evidence supporting the role of dietary probiotics in gut microbiota modulation and chronic disease mitigation [23,24]. The current investigation focuses on screening *L. plantarum* strains with optimal fermentation characteristics and safety profiles for FAJ production, while employing 16S rRNA sequencing to evaluate their impact on obese gut microbiota composition during in vitro fermentation. This integrated approach not only advances the development of functional fermented beverages but also provides novel insights into the mechanisms underlying probiotic-mediated metabolic regulation by modulating intestinal microbiota.

## 2. Materials and Methods

### 2.1. Experimental Strains

Four *L. plantarum* strains (designated 1–4) were employed for this investigation, having been isolated and purified from fermented yogurt by our research group. Prior to fermentation experiments, all four strains of *L. plantarum* were cultured to a concentration of 10^8^ CFU/mL. Subsequently, 0.1 mL aliquots of each culture were inoculated into 10 mL of Man–Rogosa–Sharpe (MRS, Qingdao Hi-tech Industrial Park Hope Biotechnology Co., Ltd., Qingdao, China) broth (containing peptone, beef extract, yeast extract, glucose, dipotassium phosphate, ammonium citrate, sodium acetate, magnesium sulfate, manganese sulfate, and Tween 80) and passaged twice under 37 °C conditions with 18 h intervals. These resulting cultures were then employed in subsequent fermentation experiments.

### 2.2. Characterization of the Growth of L. plantarum

#### 2.2.1. Growth Characteristics of *L. plantarum*

A total of 0.1 mL of *L. plantarum* (10^8^ CFU/mL) cultured in 2.1 was inoculated into 10 mL of MRS liquid medium and cultured in a shaker (Yitong Electronics Co., Ltd., Shenzhen, China) (37 °C, 180 r/min) for 18 h. After revival, the strain was transferred to sterile MRS medium and maintained at 37 °C for one day. Growth progression was tracked through spectrophotometric OD_600nm_ measurements taken every two hours, enabling growth curve plotting [25].

#### 2.2.2. Growth Characteristics of *L. plantarum* in FAJ

The above activated strains were transferred to FAJ and incubated at 37 °C for 24 h. Growth kinetics were monitored by measuring optical density (OD) at 600 nm every 2 h using a microplate reader (Nanjing DeTie Laboratory Equipment Co., Ltd., Nanjing, China) to plot the growth curve.

### 2.3. Metabolic Activity

#### 2.3.1. Analysis of Biogenic Amine Production Capability by *L. plantarum*

Following the methodology of Sang et al. [26], four strains of *L. plantarum* were initially cultured in 5 mL of MRS broth at 37 °C for 24 h. Subsequently, 100 µL of each culture was transferred into fresh 5 mL MRS broth and incubated under the same conditions for an additional 24 h. The resulting cultures were then inoculated (100 µL) into 5 mL of MRS broth supplemented with precursor compounds, followed by 37 °C incubation for 48 h to facilitate metabolite production.

For sample preparation, 1 mL of the final culture was combined with 9 mL of 10% (*w*/*v*) trichloroacetic acid (TCA). After thorough vortexing, the solution was incubated at 4 °C for 2 h to precipitate the proteins. Subsequent centrifugation at 3000× *g* (4 °C, 10 min) yielded a supernatant from which 400 µL was taken for derivatization. The derivatization process involved the sequential addition of 80 µL of 2 M NaOH, 120 µL of a saturated NaHCO_3_ solution, and 800 µL of dansyl chloride reagent. The reaction mixture was then heated at 45 °C for 40 min in a water bath, followed by termination with 50 µL of ammonia and a 30 min incubation at room temperature. The mixture was treated with 550 µL of acetonitrile, followed by centrifugation (3000× *g*, 4 °C, 5 min). After centrifugation, the liquid phase underwent dual filtration using 0.22 µm membranes prior to HPLC (Agilent 1260, Agilent Technologies, Santa Clara, CA, USA) examination. The contents of putrescine, cadaverine, histamine, and tyramine produced by the strain were determined. All experiments were conducted in triplicate.

#### 2.3.2. Hemolytic Activity Assay

For β-hemolysis analysis, 5% (*v*/*v*) sheep blood (No. TX0030, Beijing Solarbio Science & Technology Co., Ltd., Beijing, China) was added to MRS agar. The plates were incubated at 37 °C for 48 h, followed by cold shock treatment at 4 °C for 24 h. Hemolytic activity was determined by observing clear zones of lysis around bacterial colonies on the blood-supplemented MRS agar plates. For positive validation, *Virgibacillus halodenitrificans* was included in the assay [27].

#### 2.3.3. Analysis of Biofilm Formation by *L. plantarum*

The biofilm-forming capacity of *L. plantarum* was evaluated using a standardized 96-well microtiter plate assay according to the method of Zhu et al. [28], with minor modifications. Bacterial cultures were adjusted to an OD of 0.4 at 600 nm (OD_600nm_), and 200 μL aliquots were dispensed into sterile 96-well polystyrene plates, with sterile growth medium serving as the negative control and *Staphylococcus aureus* ATCC 6538 as the positive control. Static incubation of the plates was carried out at 37 °C for 48 h, with eight replicate wells per strain. Following incubation, planktonic growth was assessed by measuring OD_600nm_. The supernatant was then carefully removed, and the wells were gently washed three times with 250 μL of 1× PBS to remove non-adherent cells. The remaining adherent biofilms were immobilized with 200 μL of absolute methanol for 15 min at room temperature. Following methanol removal, the plates were air-dried at 60 °C for 15 min with the lids removed. The biofilms underwent a 15 min incubation with a 0.1% *w*/*v* crystal violet solution (200 μL per well) under ambient conditions. After staining, they were thoroughly rinsed with ultrapure water (3–5 times) to remove any excess dye. The plates were dried again at 60 °C for 30 min, and the bound dye was solubilized in 200 μL of 33% (*v*/*v*) glacial acetic acid under constant agitation for 20 min. Biofilm formation was quantified by measuring the absorbance of the solubilized crystal violet at 590 nm (OD_590nm_) using a microplate reader (Nanjing DeTie Laboratory Equipment Co., Ltd., Nanjing, China).

The cutoff value (OD_c_) for biofilm formation was defined as the mean OD_590nm_ of the negative control wells plus three times the standard deviation. Strains were classified as follows: non-biofilm formers (OD_590nm_ ≤ OD_c_), weak biofilm formers (OD_c_ < OD_590nm_ ≤ 2 × OD_c_), moderate biofilm formers (2 × OD_c_ < OD_590nm_ ≤ 4 × OD_c_), or strong biofilm formers (OD_590nm_ > 4 × OD_c_). Each experiment was performed in eight technical replicates and repeated independently five times to ensure reproducibility.

#### 2.3.4. Preparation of Apple Juice for Fermentation

The fermentation substrate consisted of Fuji apples sourced from Dalian. After thorough washing and coring, apples were cut into 4 × 4 cm pieces before pressing in a juicer (HX-PB 956, Blenders, Oaks Group Co., Ltd., Ningbo, China) for 5 min. Following filtration through an 80-mesh sieve, 0.08% pectinase and 0.8% cellulase were added to the crude juice. This mixture underwent enzymatic hydrolysis at 55 °C for 2 h, subsequent sterilization at 90 °C for 15 min, and final storage at −20 °C until needed.

### 2.4. Fermentation of Apple Juice

The second-generation bacterial culture (Section 2.1) was used as the seed inoculum, with 1% (*v*/*v*) transferred to 10 mL of apple juice. Fermentation proceeded at 37 °C until achieving a concentration of 10^8^ CFU/mL viable cells, measured via the pour plate method. Subsequently, 9 mL (3% *v*/*v*) of the seed culture, with a density of 10^8^ CFU/mL, was added to 300 mL of pasteurized apple juice. The juice had previously been treated at 80 °C for 15 min and then cooled to 25 °C. The fermentation proceeded for 24 h under 37 °C conditions [21,29]. Finally, the FAJ samples were preserved in −20 °C storage pending analysis.

### 2.5. Volatile Flavor Analysis Using Electronic Nose

The volatile flavor profiles were analyzed using a PEN-3 electronic nose system (E-nose) coupled with pattern recognition software (Win-Muster 1.6.2) for juice samples stored at 4 °C. For each analysis, 20 mL of FAJ was precisely measured in triplicate. The measurement vials were sealed with three layers of plastic film and equilibrated at ambient conditions for half an hour prior to analysis. The sensor array was purged with clean air for 120 s for baseline correction, followed by sample gas aspiration into the E-nose using a vacuum pump at a constant flow rate of 0.3 L/min. The detection phase lasted 150 s [30].

### 2.6. In Vitro Colonic Fermentation

Four obese individuals (age range: 24–32 years; BMI > 28, meeting obesity criteria) provided stool samples for analysis. All participants had no recent history of gastrointestinal disorders or antibiotic use and had maintained stable dietary habits for at least three months prior to sampling. All participants provided written informed consent, and the study protocols were approved by the institutional ethics committee (Approval No.: DLOU20250002). The non-invasive fecal collection procedure posed no risk to donors, and no human experimentation was conducted. Following a modified protocol based on Wu et al. [31], each sample was processed by homogenizing 1.0 g of feces in 10 mL of sterile PBS (pH 7.2). After vigorous vortexing, the mixture underwent centrifugation (600× *g*, 5 min, 4 °C) to eliminate particulate matter. The resulting supernatant was thoroughly mixed, reconstituted in phosphate-buffered saline, and cryoprotected by adding sterile glycerol (40% *v*/*v*) until reaching a final concentration of 20% before storage at −80 °C.

For the in vitro fermentation experiments, FAJ preparation adhered to published methodologies [32]. Briefly, the apple juice was PBS-dissolved, sterile-filtered (0.22 μm), and incorporated into an autoclaved basal medium as the exclusive carbon source (5.0 mg/mL final concentration). The nutrient medium composition was formulated with the following (per liter): peptone (2.0 g), yeast extract (2.0 g), hemin (20 mg), L-cysteine hydrochloride (500 mg), bile salts (500 mg), sodium chloride (100 mg), potassium dihydrogen phosphate (40 mg), dipotassium hydrogen phosphate (40 mg), magnesium sulfate (10 mg), calcium chloride (10 mg), sodium bicarbonate (2 g), Tween 80 (2 mL), 1% resazurin solution (1 mL), and vitamin K (10 μL).

Prior to incubation, sterile tubes containing 5.0 mL of freshly prepared medium were inoculated with 1.0 mL of the defrosted microbial solution using aseptic technique. Anaerobic fermentation proceeded at 37 °C for 96 h, with blank controls containing no FAJ. Post-fermentation, samples were centrifuged (8000× *g*, 15 min) and supernatants were harvested for subsequent gut microbiota analysis.

### 2.7. 16S rRNA Profiling

The gut microbiota profiling was analyzed by Shanghai Majorbio Bio-pharm Technology Co., Ltd., (Shanghai, China), following the same methodological approach as outlined in our previous study [33]. Briefly, bacterial genomic DNA was extracted using the E.Z.N.A.^®^ Soil DNA Kit (Tiangen Biotech Co., Ltd., Beijing, China) and quantified with a NanoDrop spectrophotometer (Thermo Fisher Scientific, Waltham, MA, USA). The V3-V4 region of the 16S rRNA gene was amplified using barcoded primers 338F/806R. The NEXTFLEX Rapid DNA-Seq Kit (Bioo Scientific Corporation, Austin, TX, USA) was employed to construct sequencing libraries, which were then analyzed using paired-end sequencing (2 × 300 bp) on an Illumina MiSeq system. The clustering of operational taxonomic units was grouped at 97% sequence similarity using Uparse (v7.0). Taxonomic classification was performed using the RDP Classifier (v2.2) on the 16S rRNA database with a confidence threshold of 0.7.

### 2.8. Statistical Analysis

Each experiment incorporated at least three biological replicates for reproducible and statistically reliable results. Data are presented as mean ± standard deviation (SD). Statistical analyses were conducted using OriginPro 2024 (OriginPro 2024 SR1, 10.1.0.178, OriginLab Corporation, Northampton, MA, USA). A one-way analysis of variance (ANOVA) followed by Tukey’s multiple comparison test was performed, with *p* < 0.05 considered statistically significant. Using the software PICRUSt2 (http://huttenhower.sph.harvard.edu/galaxy (accessed on 13 March 2024)), a number of functions were predicted for Metacyc based on PICRUSt2, and subsequent analysis of these pathways by https://metacyc.org/ (accessed on 13 March 2024). Networkx (version v1.11) software for co-occurrence network analysis and visual presentation.

## 3. Results and Discussion

### 3.1. Safety Assessment of L. plantarum

#### 3.1.1. Qualitative Analysis of Biogenic Amine Production

Biogenic amines (BAs) are a group of low-molecular-weight nitrogenous organic compounds derived from amino acid decarboxylation or amination. These compounds are commonly found in animal-derived products, plants, and protein-rich fermented foods. Excessive BA accumulation in the human body may lead to adverse physiological effects, including nausea, respiratory distress, flushing, palpitations, headaches, and hypertension or hypotension. Furthermore, BAs contribute to food spoilage and may exhibit toxic effects at elevated concentrations [34]. The toxicity threshold of BAs varies depending on individual tolerance, detoxification capacity, and genetic susceptibility. International regulatory agencies have established BA limits primarily for aquatic products and alcoholic beverages, with a focus on histamine and tyramine levels [26].

Lactic acid bacteria (LAB) play a crucial role in fermented foods by enhancing flavor, texture, and nutritional value. However, the potential of probiotic strains to generate harmful BAs through metabolic pathways remains insufficiently investigated. In LAB, proteases and peptidases catalyze the degradation of milk proteins, releasing free amino acids, such as tyrosine, alanine, and histidine, which serve as precursors for BAs synthesis. Notably, certain Gram-positive and Gram-negative bacteria, as well as yeasts in fermented products, have been reported to possess amino acid decarboxylase activity. Among LAB, species of *Lactococcus*, *Lactobacillus*, and *Leuconostoc* are associated with the production of histamine, tyramine, and putrescine [35]. Therefore, monitoring BA levels is essential for ensuring the safety of probiotic strains.

Four *L. plantarum* strains exhibiting the capacity to produce biogenic amines (BAs)—putrescine, cadaverine, histamine, and tyramine—were evaluated (Table 1). The results demonstrated that none of the strains produced detectable levels of histamine or tyramine. Regarding putrescine and cadaverine, all four strains exhibited low production levels; however, the comparative analysis revealed that *L. plantarum*-1 generated significantly lower amounts of putrescine and total BAs than the other three strains, suggesting its superior safety profile.

#### 3.1.2. Hemolytic Activity Assessment of *L. plantarum*

Hemolysis refers to the ability of microorganisms to lyse red blood cells by disrupting their membranes, a virulence mechanism employed by certain pathogens to invade host tissues. Since probiotics may pose potential risks to immunocompromised individuals, pregnant women, children, or under specific conditions (e.g., allergic reactions or drug interactions), safety evaluations—including hemolytic activity assays—are essential prior to their widespread application [36,37].

**Figure 1:**
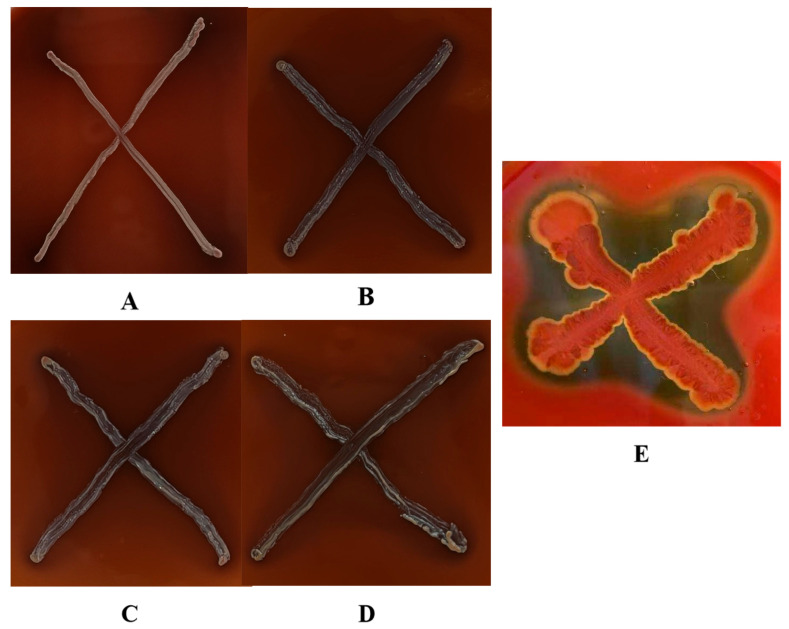
Haemolytic activity of strains of *L.plantarum*: (**A**–**D**) haemolytic activity of *L. plantarum* 1–4; and (**E**) haemolytic activity of *Virgibacillus halodenitrificans*.

In this study, hemolytic activity was qualitatively analyzed on MRS agar supplemented with 5% defibrinated sheep blood. Strains were categorized as β-hemolytic (showing clear zones indicative of potential pathogenicity) or γ-hemolytic (showing no hemolysis). Figure 1 demonstrates that while *Virgibacillus halodenitrificans* (reference strain) exhibited pronounced β-hemolysis, all *L. plantarum* isolates displayed γ-hemolytic characteristics. The complete absence of β-hemolytic activity in *L. plantarum* strains indicates their non-hemolytic nature, affirming their safety potential as fermentation starters for apple juice [27].

#### 3.1.3. Analysis of Biofilm Formation Capacity in *L. plantarum*

Biofilms exhibit unique physical stability, surface-sensing capabilities, and internal microenvironmental gradients, enabling them to effectively maintain moisture balance and nutrient cycling within microbial communities [38,39]. This highly organized structure not only provides a physical protective barrier for microorganisms but also optimizes ecological niche allocation among beneficial bacteria by facilitating interspecies interactions. Numerous studies have demonstrated that probiotic strains can form biofilms on both abiotic (e.g., glass or polystyrene) and biotic surfaces, a trait considered advantageous and highly strain-dependent. Biofilm formation enhances probiotic colonization and persistence on host mucosal surfaces, inhibits the growth, surface adhesion, and biofilm formation of foodborne pathogens, and suppresses fungal proliferation. As a result, probiotic-derived biofilms have emerged as a novel and safe antimicrobial strategy against pathogenic bacteria [40,41].

Furthermore, research indicates that biofilm formation improves probiotic resilience under adverse environmental conditions, including stress resistance, survival rates, enzymatic activity, and colonization efficiency. These attributes are closely linked to the technical performance of probiotic strains and their ability to exert beneficial effects [40].

In this study, the biofilm-forming capacity of *L. plantarum* strains was quantitatively evaluated (Table 2). All four tested *L. plantarum* strains demonstrated measurable biofilm production as calculated by the standard formula. Notably, *L. plantarum*-1 exhibited the highest OD value in biofilm formation, with results closely approximating the positive control *Staphylococcus aureus*. This superior biofilm-forming capability suggests that *L. plantarum*-1 may possess enhanced potential for intestinal colonization compared to the other tested strains.

The ability of *L. plantarum* to form biofilms underscores its potential as a probiotic with enhanced environmental adaptability and competitive exclusion of pathogens. Given that biofilm formation correlates with improved stress tolerance and colonization efficiency, these strains may exhibit superior performance in industrial applications, such as functional food production and gut microbiota modulation [42].

### 3.2. Characterization of the Growth of L. plantarum

**Figure 2:**
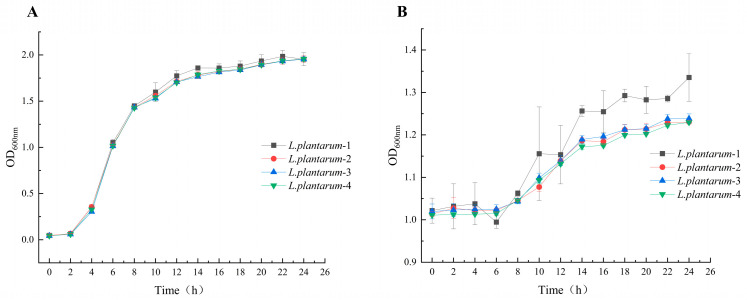
Growth characteristic curves of *L.plantarum* in MRS broths (**A**) and in apple juice (**B**). Three biologically independent replicates were performed to construct growth curves for each isolate.

The growth kinetics of *L. plantarum* in MRS broth were monitored by measuring OD at 2 h intervals (Figure 2A). An initial lag phase (0–2 h) was observed, during which bacterial cells adapted to the new environment with no significant OD increase. Exponential growth commenced after 2 h of inoculation, followed by a progressive decline in growth rate after 8 h. The stationary phase was reached at 14 h, corresponding to the maximum biomass yield.

During fermentation, samples were collected every 2 h to monitor changes in OD (Figure 2B). The OD values of the apple juice exhibited an increasing trend over time, consistent with growth patterns reported in previous studies [43]. In the initial fermentation phase (0–8 h), the OD values remained relatively stable across all the experimental groups, indicating that the probiotic bacteria were undergoing an environmental adaptation phase. During this stage, the bacterial cells initiated metabolic reprogramming to synthesize essential enzymes and metabolites but had not yet entered the proliferative phase.

After 8 h of fermentation, the bacterial population entered the logarithmic growth phase (8–16 h), characterized by a significant increase in OD values, exponential growth in viable cell counts, and a marked enhancement in metabolic activity. By 16 h, the total viable count reached its peak, suggesting that the culture system had achieved maximum cell density. Subsequently, the viable cell numbers stabilized, and the OD values increased only marginally.

The four *L. plantarum* strains exhibited comparable growth kinetics in standard medium (Figure 2A), while showing distinct growth profiles in apple juice, with *L. plantarum*-1 demonstrating significantly superior growth performance and biomass accumulation (Figure 2B). This strain-specific enhancement in apple juice suggests *L. plantarum*-1 possesses specialized metabolic adaptations for efficient nutrient utilization in fruit-based substrates, highlighting its potential suitability for apple juice fermentation applications. The observed growth differences between standard and application-specific media underscore the importance of evaluating strain performance under conditions relevant to their intended use.

### 3.3. Response of the Electronic Nose to Signals of Flavor Substances in FAJ from Different Strains

**Figure 3:**
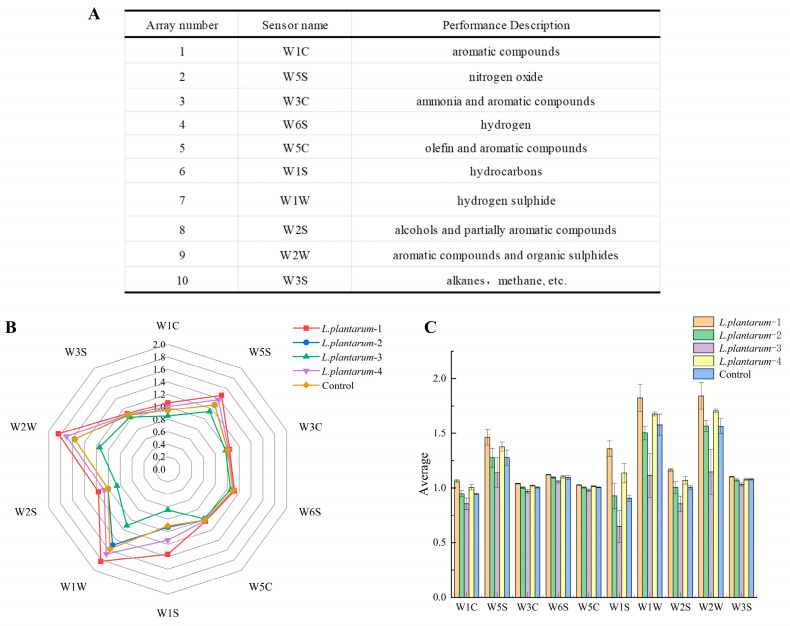
Radar analyses of apple juice fermentation by four strains of bacteria: (**A**) performance of each sensor; (**B**) radar plot of response values; and (**C**) histogram of mean response values. Three independent experimental replicates were conducted.

The electronic nose (E-nose) system mimics human olfaction through ten uniquely responsive metal oxide semiconductor (MOS) sensors arranged in an array, the substances corresponding to each sensor of the electronic nose are shown in Figure 3A. To compare the volatile organic compound profiles in apple juice produced through fermentation with four distinct *L. plantarum* strains, relative resistivity (G/G0) measured at 58–60 s was plotted as a radar diagram (Figure 3B). The radar plot consists of 10 axes, each representing a specific sensor, with the radial distance indicating the sensor’s sensitivity to the corresponding sample. Among the sensors, five (W1W, W2W, W1S, W2S, and W5S) exhibited the highest responses to sulfur-containing compounds, alcohols, methyl groups, organic sulfides, and nitrogen oxides, respectively, with notable differences observed among the samples. These compounds—particularly esters, aldehydes, and alcohols—are known to dominate apple juice aroma, followed by ketones [44]. The remaining five sensors (W1C, W3C, W6S, W5C, and W3S) detected distinct volatile components—including aromatics, nitrogen-based molecules, and hydrocarbons of varying chain lengths—which further shaped the overall flavor profile, though their responses showed less variation across the different *L. plantarum* strains. Furthermore, Figure 3C demonstrates that the *L. plantarum*-1-fermented juice displayed the most pronounced flavor profile among the tested samples. This strain was consequently selected for further in vitro fermentation studies based on its optimal combination of functional properties: minimal biogenic amine production, demonstrated biofilm formation capacity, *γ*-hemolytic safety profile, and significant flavor enhancement potential in FAJ.

### 3.4. Effect of FAJ on Gut Microbiota

#### 3.4.1. FAJ-Induced Changes in Gut Microbial Composition at the Phylum Level

**Figure 4:**
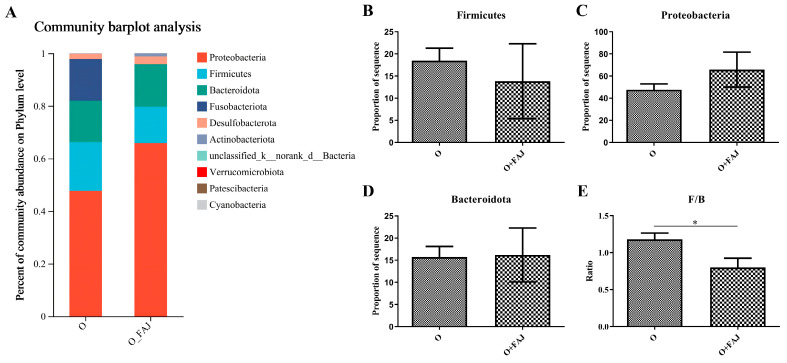
Effect of fermented apple juice on gut flora phylum levels: (**A**) heatmap of phylum level; (**B**–**D**) relative abundance of Firmicutes, Proteobacteria, and Bacteroidota; and (**E**) ratio of Firmicutes/Bacteroidota; “O” denotes the obesity group, “O+FAJ” denotes fermented apple juice group; * denotes significant difference.

At the phylum level, Proteobacteria, Firmicutes, and Bacteroidota constituted the major bacterial taxa in obese and FAJ-treated subjects, representing more than 80% of the total microbial community (Figure 4A), collectively accounting for over 80% of the total relative abundance. Notably, after FAJ intervention, their combined relative abundance increased to 90%.

As shown in Figure 4B,D, although FAJ intervention did not significantly alter the individual abundances of Firmicutes, Proteobacteria, or Bacteroidota (*p* > 0.05), the Firmicutes-to-Bacteroidota (F/B) ratio exhibited a statistically significant decrease (*p* < 0.05, Figure 4E). This finding holds physiological significance, since prior research has demonstrated that increased F/B ratios associate positively with obesity—Firmicutes are generally more abundant in obese individuals, while Bacteroidota exhibit an opposite pattern [33,45].

Accumulating evidence suggests that a reduced F/B ratio is closely associated with body weight regulation. For instance, Lee et al. [46] demonstrated that *Lactobacillus paracasei* BEPC22 and *L. plantarum* BELP53 administration prevented excessive weight and fat increases in murine models receiving high-fat diets, with F/B ratio reduction as the underlying mechanism. Similarly, comparative 16S rRNA sequencing of fecal samples revealed a markedly elevated Firmicutes-to-Bacteroidetes ratio in individuals with metabolic dysfunction-associated steatotic liver disease (MASLD) compared to healthy controls [47]. These findings collectively suggest that FAJ juice can exert anti-obesity effects by modulating the F/B ratio, highlighting its potential in obesity prevention and management.

#### 3.4.2. FAJ-Induced Changes in Gut Microbial Composition at the Genus Level

**Figure 5:**
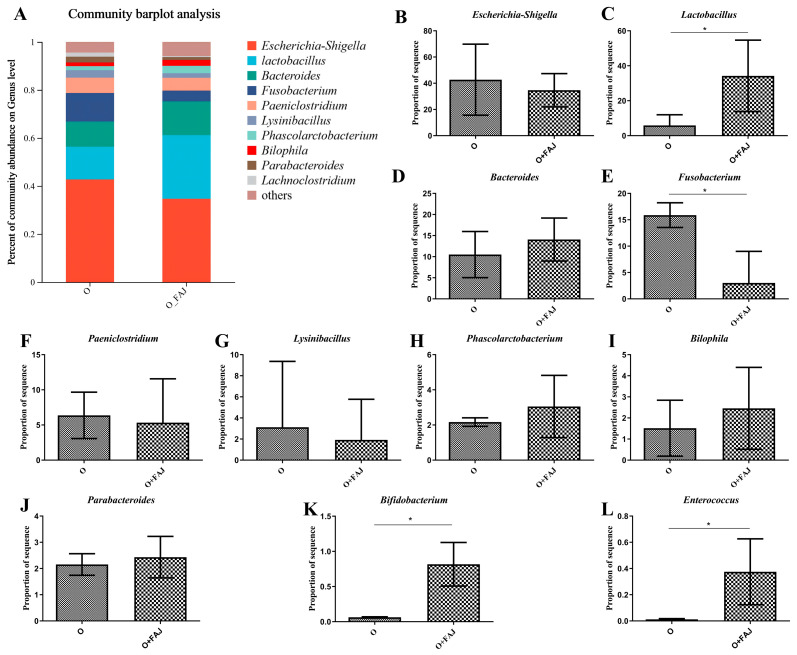
Effect of fermented apple juice on gut flora genus levels: (**A**) heatmap of genus level; (**B**–**L**) the relative abundance of *Escherichia-Shigella*, *Lactobacillus*, *Bacteroides*, *Fusobacterium*, *Paeniclostridium*, *Lysinibacillus*, *Phascolarctobacterium*, *Bilophila*, *Parabacteroides*, *Bifidobacterium*, and *Enterococcus*; “O” denotes obese group, “O+FAJ” denotes fermented apple juice group, and * denotes significant difference.

To examine how FAJ affects the gut microbiota in obese individuals, we assessed the ten most prevalent bacterial genera (Figure 5). Notably, potential opportunistic pathogens, such as *Fusobacterium* and *Escherichia-Shigella,* were significantly elevated in obese individuals, with *Fusobacterium* showing a marginal reduction after FAJ intervention (*p* > 0.05).

Previous studies have demonstrated that *Lysinibacillus*, which was significantly reduced by FAJ in our study (*p* < 0.001), can degrade aspirin and counteract its anticancer effects mediated through gut microbiota modulation [48]. Similarly, *Bilophila*, a microorganism associated with high-fat diets and bile acid metabolism [49], exhibited a marked decrease following intervention (*p* < 0.001). In contrast, beneficial genera including *Parabacteroides* and *Bifidobacterium* showed significant increases (*p* < 0.001). *Parabacteroides* species, such as *P. goldsteinii* and *P. distasonis,* are recognized as potential probiotics for alleviating obesity-related metabolic dysfunction [50], while *Bifidobacterium* is known to enhance intestinal barrier function and immune response [51].

Additionally, we observed modest, though non-significant, increases in *Lactobacillus*, which modulates gut microbiota and host health [52]; *Bacteroides*, which plays a critical role in obesity prevention [53]; *Phascolarctobacterium*, a key regulator of body weight and microbial balance [54]; and *Enterococcus*, which promotes thermogenesis in brown adipose tissue to reduce fat accumulation [55]. These findings collectively indicate that FAJ modulates gut microbiota composition by suppressing conditionally pathogenic and fat-associated bacteria while promoting beneficial genera. While this study is limited to in vitro experiments and cannot fully replicate the complexity and dynamic interactions of the gut microbiota in vivo, our findings provide preliminary insights into potential modulations of microbial abundance under physiological conditions. The results consistently demonstrate that *L. plantarum*-1 exhibits promising characteristics that may contribute to obesity alleviation, though further in vivo validation remains essential.

#### 3.4.3. MetaCyc Pathway Analysis

**Figure 6:**
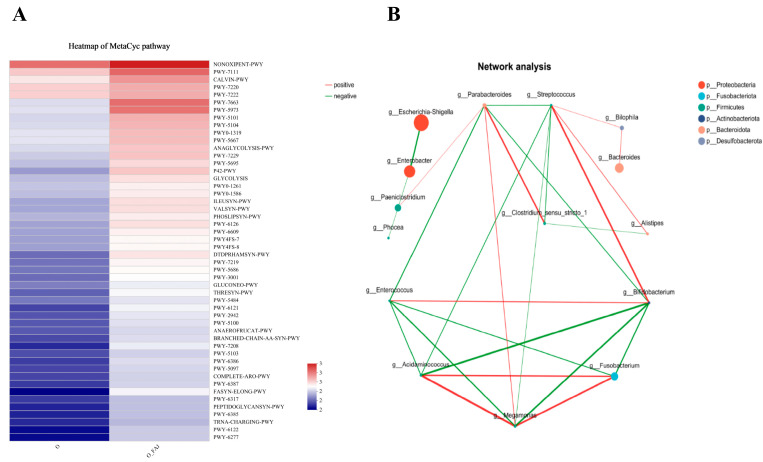
Functional analysis and species network correlation graph: (**A**) MetaCyc pathway heatmap; (**B**) Network analysis graph; note: The default display of the graph shows species with *p* < 0.05; the size of the nodes in the graph indicates the abundance size of the species, and different colors indicate different species. The color of the connecting lines indicates a positive and negative correlation, with red indicating a positive correlation, and green a negative correlation; the thickness of the lines indicates the correlation coefficient. The thickness of the line indicates the size of the correlation coefficient, the thicker the line, the higher the correlation between the species. A greater number of links indicates stronger relationships among different species.

The MetaCyc database analysis revealed the functional abundance distribution across different samples (Figure 6A), visually representing the predominant metabolic pathways. A color gradient heatmap illustrates variations in pathway abundances among samples/groups. The right panel highlights pathways primarily associated with glycolysis, including NONOXIPENT-PWY, PWY-7111, CALVIN-PWY, PWY-7663, PWY-5973, P42-PWY, ILEUSYN-PWY, VALSYN-PWY, PWY-6126, and DTDPRHAMSYN-PWY. Following FAJ intervention, all glycolytic pathways exhibited significant enrichment, with NONOXIPENT-PWY being particularly prominent. As a canonical glycolytic pathway, NONOXIPENT-PWY is widely utilized by organisms for glucose and related sugar catabolism. Enhanced glycolytic activity may promote white adipose tissue browning, which not only counteracts high-fat diet-induced obesity through lipolysis but also improves glucose uptake and insulin sensitivity [56]. The FAJ-induced upregulation of glycolytic functions in obese samples suggests accelerated intestinal sugar-to-energy conversion, contributing to obesity mitigation. These results further support the potential of FAJ in obesity prevention and intervention.

#### 3.4.4. Network Correlation Analysis

We constructed a species correlation network using the top 20 most abundant genera to elucidate the interactions among dominant microbial species, with Spearman’s correlation coefficients calculated to assess interspecies relationships. In the network analysis of FAJ-treated gut microbiota (Figure 6B), “degree” represents the number of connections per node. At the same time “clustering coefficient” reflects the interconnectivity among adjacent nodes (ranging from 0, no connections, to 1, complete interconnectivity). Key genera exhibited prominent network roles, with *Streptococcus*, *Parabacteroides*, *Enterococcus*, *Acidaminococcus*, *Megamonas*, and *Bifidobacterium* having degrees of 7, 6, 5, 5, 6, and 6. Notably, *Streptococcus* showed significant positive correlations with *Bifidobacterium*, while *Acidaminococcus* correlated positively with *Megamonas* but negatively with *Bifidobacterium*. *Bifidobacterium* displayed positive associations with *Enterococcus* but negative correlations with *Parabacteroides*, *Acidaminococcus*, and *Megamonas*. Conversely, *Megamonas* was positively linked to *Acidaminococcus* and *Parabacteroides* but negatively to *Enterococcus* and *Streptococcus*.

The observed correlations align with known functional roles of these taxa. *Bifidobacterium*, a keystone genus in gut homeostasis [57], antagonized *Acidaminococcus* and *Megamonas*—both implicated in obesity [58]. *Enterococcus*, a commensal with probiotic potential (e.g., E. faecium), contributes to immunomodulation and fermented food applications [59]. *Streptococcus* may mitigate obesity via exopolysaccharide production [60]. The intervention with fermented apple juice appeared to promote beneficial taxa (*Bifidobacterium* and *Streptococcus*) while suppressing obesity-associated genera (*Acidaminococcus*, and *Megamonas*), suggesting a microbiota-mediated anti-obesity mechanism.

## 4. Conclusions

This study evaluated the safety characteristics and fermentation flavor profiles of four *L. plantarum* strains. Based on the assessment results, *L. plantarum*-1 exhibited low biogenic amine production, no hemolytic activity, the ability to form probiotic biofilms, and favorable flavor-enhancing properties. The results showed that the strain had the best characteristics and was used as the strain for subsequent fermentation experiments.

In the fermented apple juice, inorganic sulfides, methyl compounds, alcohols, aldehydes/ketones, and nitrogen oxides were identified as the primary contributors to flavor. Furthermore, fermentation with *L. plantarum*-1 significantly reduced the Firmicutes/Bacteroidota ratio in obese microbiota while increasing the abundance of *Enterococcus*, *Parabacteroides*, and *Bifidobacterium*, indicating a modulatory effect on gut microbial composition. Additionally, the fermented apple juice enhanced glycolytic function, suggesting a potential mechanism for obesity alleviation.

These findings demonstrate the application potential of *L. plantarum*-1 in fermented apple juice production. The study not only expands the scope of functional fermented food development but also provides a novel approach for creating health-promoting apple-based beverages. Moreover, it offers critical theoretical insights into the regulatory effects of probiotic-fermented apple juice on human gut microbiota. However, certain limitations should be acknowledged. The laboratory-based fermentation approach, though useful, may fall short of encompassing the true biological intricacies of the in vivo gut ecosystem. Additionally, the limited demographic diversity of the samples may restrict the generalizability of the findings. Furthermore, the absence of comparative studies with well-characterized reference strains hinders the definitive assessment of *L. plantarum*-1′s relative efficacy. Future research should incorporate more diverse human samples, include comparisons with established bacterial strains, and employ appropriate in vivo validation models to strengthen these preliminary observations and facilitate practical applications.

## Figures and Tables

**Table 1:** Content of biogenic amines in four strains of *L. plantarum*.

Strains	mg/L				
	Putrescine	Cadaverine	Histamine	Tyramine	Total BAs
*L. plantarum*-1	30.55 ± 1.20 ^d^	0.59 ± 0.25 ^a^	n.d.	n.d.	31.14 ± 1.04 ^d^
*L. plantarum*-2	33.52 ± 1.30 ^c^	0.30 ± 0.02 ^b^	n.d.	n.d.	33.82 ± 1.28 ^c^
*L. plantarum*-3	47.28 ± 1.04 ^b^	0.15 ± 0.12 ^c^	n.d.	n.d.	47.43 ± 1.16 ^b^
*L. plantarum*-4	50.74 ± 1.00 ^a^	0.61 ± 0.27 ^a^	n.d.	n.d.	51.35 ± 0.73 ^a^

Data are presented as mean ± SD, *n* = 3; within a given column, distinct superscript letters (a, b, c, and d) denote statistically significant variations (*p* < 0.05); and histamine and tyramine were not detected in any of the samples. n.d. stands for not detected.

**Table 2:** Biofilm measurement results.

Strain Number	OD_600nm_	OD_590nm_
Control	−0.545 ± 0.229	−0.029 ± 0.005
*Staphylococcus aureus* ATCC6538	0.431 ± 0.101 ^a^	0.300 ± 0.038 ^a^
*L. plantarum*-1	0.360 ± 0.108 ^a^	0.230 ± 0.004 ^b^
*L. plantarum*-2	0.420 ± 0.142 ^a^	0.199 ± 0.017 ^b^
*L. plantarum*-3	0.356 ± 0.090 ^a^	0.206 ± 0.022 ^b^
*L. plantarum*-4	0.372 ± 0.059 ^a^	0.219 ± 0.009 ^b^

Results are expressed as mean ± standard deviation, *n* = 5; within a given column, distinct superscript letters (a, b) denote statistically significant variations (*p* < 0.05).

## Data Availability

The original contributions presented in this study are included in the article. Further inquiries can be directed to the corresponding author.
